# Maternal Obesity Programs Adipogenic Commitment in Neonatal Mesenchymal Stem Cells: A Link to Redox‐Dependent FOXO1 Signaling

**DOI:** 10.1002/jcp.70178

**Published:** 2026-04-28

**Authors:** Sofía Bellalta, Erika Pinheiro‐Machado, Theo Borghuis, Jelmer Prins, Torsten Plösch, Paola Casanello, Marijke Faas

**Affiliations:** ^1^ Department of Pathology and Medical Biology, University of Groningen University Medical Center Groningen Groningen the Netherlands; ^2^ Department of Obstetrics, School of Medicine Pontificia Universidad Católica de Chile Santiago Chile; ^3^ Department of Obstetrics and Gynaecology, University of Groningen University Medical Center Groningen Groningen the Netherlands; ^4^ Perinatal Neurobiology Research Group, School of Medicine and Health Sciences Carl von Ossietzky University of Oldenburg Oldenburg Germany; ^5^ Department of Neonatology, School of Medicine Pontificia Universidad Católica de Chile Santiago Chile

**Keywords:** adipogenesis, maternal obesity, mesenchymal stem cells, oxidative stress, redox balance

## Abstract

Maternal obesity increases the risk of obesity and metabolic disease in the offspring, yet the cellular mechanisms that program adipose tissue development remain poorly understood. Mesenchymal stem cells (MSCs), the precursors of adipocytes, may represent an early target of metabolic programming during fetal development. In this study, we investigated whether maternal obesity alters stemness, redox homeostasis and adipogenic signaling through FOXO1 in neonatal MSCs. MSCs were isolated from Wharton's jelly of umbilical cords from neonates born to mothers with normal weight (NW‐MSCs, *n* = 15) or obesity (OB‐MSCs, *n* = 15). OB‐MSCs exhibited reduced stemness characteristics, including lower OCT3/4 expression and decreased clonogenic capacity. These cells also displayed increased mitochondrial superoxide levels and reduced SOD2 expression, indicating mitochondrial oxidative stress. In addition, OB‐MSCs showed increased GSH levels and decreased antioxidant enzyme response, compared to NW‐MSCs. Further, OB‐MSCs exhibited higher FOXO1 expression levels, reduced acetyl‐FOXO1 levels, and altered subcellular localization during early adipogenesis, consistent with reduced repression of the adipogenic regulator PPARγ. Finally, OB‐MSC–derived adipocytes exhibited increased PPARγ expression at later stages of differentiation. These findings suggest that maternal obesity disrupts redox balance and FOXO1 dynamics in neonatal MSCs, thereby shifting early adipogenic signaling toward enhanced adipocyte commitment. This early programming mechanism may expand the adipocyte precursor pool, potentially predisposing the progeny to higher adiposity and metabolic disorders later in life.

## 1 Introduction

Maternal obesity is associated with increased fetal growth and adiposity in the offspring (Catalano et al. 2009; Poston et al. 2016; Voerman et al. 2019). Recent studies propose that mesenchymal stem cells (MSCs), the precursors of adipocytes, isolated from neonates of mothers with obesity, exhibit enhanced adipogenic differentiation potential (Chen et al. 2016; Iaffaldano et al. 2018; Bellalta et al. 2024). These findings indicate that maternal obesity may program fetal adipose precursor cells; however, the underlying mechanisms remain unclear.

MSCs give rise to the mesodermal cell lineages and are established during early embryonic development (Ullah et al. 2015). They are present in fetal tissues, including the Wharton's jelly of the umbilical cord (La Rocca et al. 2009; Araújo et al. 2017; Guenther et al. 2022), and display fundamental stemness properties, such as self‐renewal and multipotency (La Rocca et al. 2009; Ullah et al. 2015; Hiew and Teoh 2022). These properties are strongly influenced by the cellular microenvironment and critically determine subsequent lineage commitment (Ryall et al. 2015; Chen et al. 2016).

Reactive Oxygen Species (ROS), mainly generated by the mitochondrial respiratory chain, are by‐products of cellular aerobic metabolism and include superoxide radical anion (O_2_^•‐^) and hydrogen peroxide (H₂O₂) (Schieber and Chandel 2014; Sies et al. 2017; Sies et al. 2022). Intracellular ROS levels are tightly regulated by antioxidant systems such as glutathione (GSH) (Averill‐Bates 2023), superoxide dismutase (SOD), GSH peroxidase (GPX), and catalase (CAT) (Hewitt and Degnan 2023). Impaired redox regulation leads to ROS accumulation, oxidative stress, and cellular damage (Sies et al. 2017). However, moderate ROS levels play an important role in MSC commitment toward the adipogenic lineage (Lee et al. 2009; Kojima et al. 2010; Ducluzeau et al. 2011; Tormos et al. 2011; Kanda et al. 2011; Zhang et al. 2013; Higuchi et al. 2013; Atashi et al.^2015^). This suggests that redox signaling may influence adipogenic differentiation pathways.

Adipogenesis is a multistep process controlled by transcription factors that regulate adipogenic gene expression. Forkhead box class O1 (FOXO1), a downstream target of the Insulin‐induced kinase of phosphatidylinositol 3‐kinase (PI3K) and protein kinase B (Akt) signaling pathways (Cheng and White 2011), regulates cell cycle, oxidative stress responses, and lipid metabolism during early adipogenesis (Klotz et al. 2015; Chen et al. 2019). Initially, FOXO1 represses the key adipogenic regulator Peroxisome proliferator‐activated receptor γ (PPARγ) via direct interaction (Armoni et al. 2006). Subsequently, FOXO1 releases PPARγ, enabling activation of adipogenic genes and adipocyte maturation (Chen et al. 2019; Munekata and Sakamoto 2009). FOXO1 activity and subcellular localization are modulated by post‐translational modifications (Boccitto and Kalb 2011), including acetylation and deacetylation (Qiang et al. 2010).

Sirtuins (SIRTs) are NAD+ ‐dependent histone deacetylases that act as co‐regulators in oxidative stress (Gomes et al. 2015). SIRT2, the predominant sirtuin expressed in adipocytes, is mainly localized in the cytoplasm (Gomes et al. 2015). During early commitment, SIRT2 deacetylates FOXO1, promoting its nuclear translocation and repression of PPARγ. Conversely, SIRT2 knockdown increases FOXO1 acetylation and promotes its exclusion from the nucleus (Jing et al. 2007), reducing FOXO1‐mediated repression of PPARγ and facilitating adipocyte differentiation (Dowell et al. 2003; Jing et al. 2007; Wang and Tong 2009).

Although maternal obesity has been associated with increased adipogenic potential in neonatal MSCs, the molecular pathways remain poorly understood. Because redox signaling is a key regulator of both stem cell fate and FOXO1 transcription factor activity (Klotz et al. 2015; Jing et al. 2007; Wang and Tong 2009), alterations in the redox balance of MSCs may represent an early programming mechanism of adipogenesis. Here, we hypothesize that maternal obesity alters MSCs redox state, thereby modulating the SIRT2–FOXO1 adipogenic pathway. To test this, we examined early adipogenic differentiation in Wharton's jelly–derived MSCs from neonates born to mothers with normal weight (NW‐MSCs) or obesity (OB‐MSCs).

## 2 Results

### 2.1 Wharton's Jelly‐Derived Cells Express MSC Markers and have Multipotent Potential

**Figure 1:**
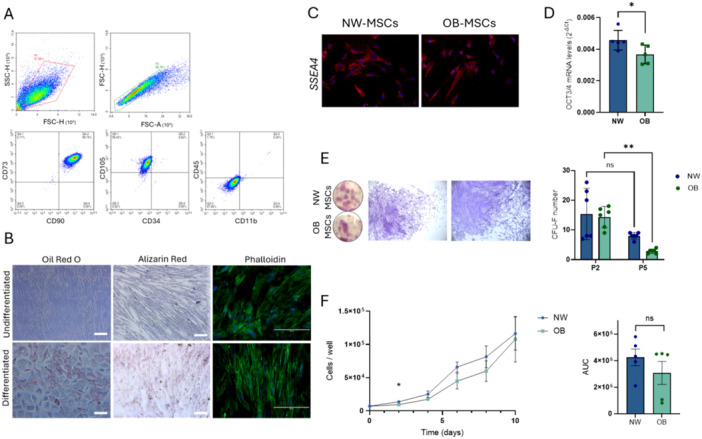
OB‐MSCs exhibit lower stemness, clonogenic capacity, and growth rate. (A) Wharton jelly‐derived mesenchymal stem cells were positive for surface markers CD73, CD90, and CD105, while negative for CD34, CD45, and CD11b in NW‐MSCs and O•B‐MSCs (*n* = 3). The gating of cells is plotted as forward scatter versus side scatter for the cell population and forward scatter versus forward scatter for singlets (top). Representative graphs show surface markers of NW‐MSCs. (B) MSCs were cultured in induction media (osteogenic and myogenic) for 14 days, and in adipogenic induction medium for 21 days. MSCs were stained for lipids (Oil Red O), calcium (Alizarin Red), and α‐SMA (phalloidin) (*n* = 3 NW‐MSCs and 3 OB‐MSCs). Representative images show induction of NW‐MSCs (Scale bars Oil Red O and Alizarin Red: 100 μm; Phalloidin: 400 μm). (C) MSCs from normal weight and obesity groups stained for Stage Specific Embryonic Antigen 4 (SSEA‐4) (scale bar: 100 μM). (D) OB‐MSCs showed lower gene expression levels of the stemness marker OCT3/4, compared to NW‐MSCs (*n* = 5). (E) Representative images of colonies per well with magnification (left) and quantification of positive colonies at day 14 (right, *n* = 6 NW‐MSCs and 6 OB‐MSCs). (F) Growth curve of cell populations (left, *n* = 5 NW‐MSCs and 5 OB‐MSCs). Area under the curve (AUC) of NW‐MSCs and OB‐MSCs growth for 10 days (right). *p < 0.05 Mann−Whitney *U* test; **p < 0.01 Tukey's range post hoc test.

Wharton's jelly tissue explants from all subjects showed outgrowth of adherent MSCs. Cells from both groups were > 88% positive for MSC markers CD73 and CD90, and CD105; and negative for hematopoietic/leukocyte markers CD11b, CD34, and CD45 (Figure 1A). There was no difference in the expression of any marker between NW‐MSCs and OB‐MSCs (Supporting Information S1: Table 1). Both NW‐MSCs and OB‐MSCs were positive for lipid accumulation (Oil red O staining) when induced for adipogenic differentiation; exhibited calcium deposit accumulation upon osteogenic differentiation (Alizarin Red staining), and displayed alpha‐smooth muscle actin fibers (α‐SMA) after myogenic differentiation (Phalloidin staining, Figure 1B).

### 2.2 OB‐MSCs Exhibit Reduced Stemness Compared to NW‐MSCs

NW‐MSCs and OB‐MSCs were positive for SSEA4. There was no difference in the fluorescence intensity between both groups (Figure 1C). However, gene expression of OCT3/4 was decreased in OB‐MSCs compared to NW‐MSCs (*p* = 0.03) (Figure 1D). NW‐MSCs formed 15.3 ± 8.6 colonies, compared with 14.3 ± 3.5 colonies in OB‐MSCs for P2. In P5, NW‐MSCs exhibited 7.6 ± 1.5, compared with 2.8 ± 0.7 colonies in OB‐MSCs. There was an effect of the passage over the number of colonies (*p* < 0.0001), with a decrease in the number of colonies in passage 2 compared to 5 only for OB‐MSCs (*p* = 0.002, Figure 1E).

### 2.3 OB‐MSCs Have a Lower Initial Growth Rate Compared to NW‐MSCs

NW‐MSCs and OB‐MSCs proliferated for 10 days from 7.000 to 116.333 ± 49.511 cells and 94.583 ± 70.732 cells, respectively. The area under the curve in NW‐MSCs was 425.400 ± 140.396, whereas it was 307.000 ± 194.664 in OB‐MSCs. This difference was not significant; however, OB‐MSCs proliferated to 9.300 ± 2.167 cells, whereas NW‐MSCs exhibited a significantly higher number of cells (13.733 ± 2.644 cells) on Day 2 (*p* = 0.01). Further, on Days 4, 6, 8, and 10, there was no difference in the number of cells between the two groups (Figure 1F).

### 2.4 OB‐MSCs Show Higher Mitochondrial Oxidative Stress Compared to NW‐MSCs in Basal State

**Figure 2:**
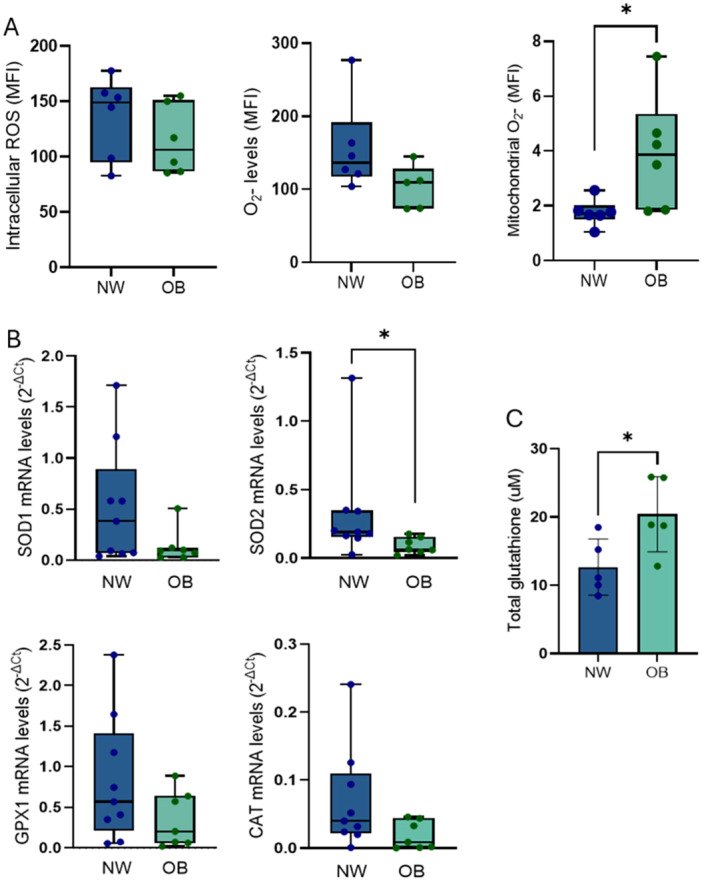
Basal redox state and antioxidant mechanisms are altered in OB‐MSCs compared to NW‐MSCs. (A) Basal ROS levels: cells were incubated with H2DCFDA for total intracellular ROS, DHE for total O_2_^•‐^, or mitoSOX for mitochondrial O_2_^•‐^. (B) SOD1/2, GPX1 and CAT gene expression were measured in both groups. (C) Glutathione intracellular levels in NW‐MSCs and OB‐MSCs. *p < 0.05, Mann−Whitney *U* test, median ± range, *n* = 9 NW‐MSCs and 7 OB‐MSCs.

To evaluate basal ROS levels, we measured total intracellular ROS, total intracellular O_2_^•‐^ and mitochondrial O_2_^•‐^ in NW‐MSCs and OB‐MSCs. Total intracellular ROS and total intracellular O_2_^•‐^ levels were not different between both groups (*p* = 0.39 and *p* = 0.1, respectively). However, there was an increase in the levels of mitochondrial O_2_^•‐^ of OB‐MSCs compared to NW‐MSCs in the basal state (*p* = 0.03, Figure 2A).

### 2.5 Basal Antioxidant Mechanisms Are Altered in OB‐MSCs Compared to NW‐MSCs

We evaluated the mRNA expression of the antioxidant enzyme genes. OB‐MSCs showed lower basal mRNA expression of SOD2 compared with NW‐MSCs (*p* = 0.03), with no differences between OB‐MSCs and NW‐MSCs in the expression of SOD1, GPX1 or CAT (*p* = 0.2, 0.1 and 0.1, respectively, Figure 2B). Conversely, levels of GSH were 20.4 ± 4.9 μM in OB‐MSCs, and 12.6 ± 3.61 μM in NW‐MSC, indicating a higher redox buffering capacity in OB‐MSCs (Figure 2C). We did not find differences in the ratio of GSH/GSSG between NW‐MSCs and OB‐MSCs, which is another indicator of the GSH buffering capacity (data not shown).

### 2.6 OB‐MSCs Exhibit Lower Levels of Intracellular ROS in Response to a Pro‐Oxidative Challenge

**Figure 3:**
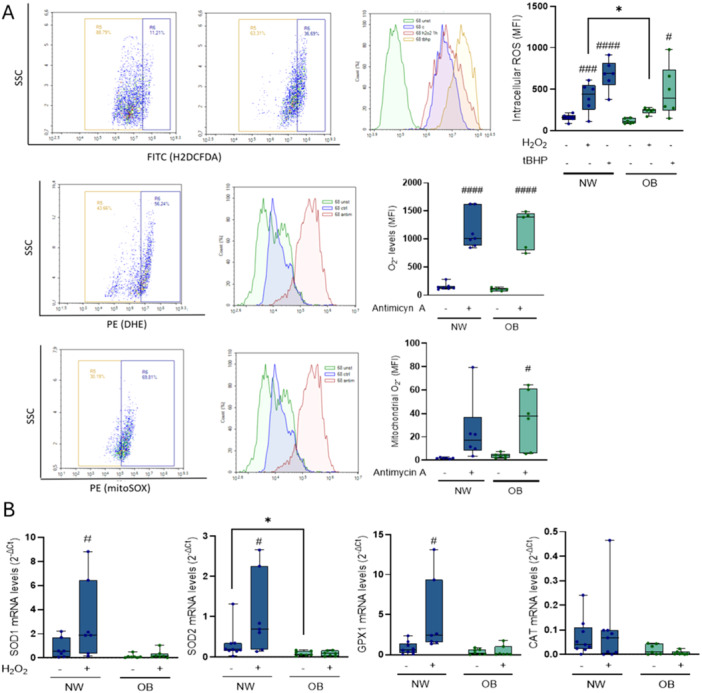
Induced ROS levels in NW‐MSCs and OB‐MSCs. (A) *Top:* MSCs were incubated with H2DCFDA after incubation with or without H₂O₂ (400 μM) or tBHP (100 μM) to assess induced ROS levels. Two‐way ANOVA: Effect of H₂O₂ (*p* = 0.0001), tBHP (*p* < 0.0001) and obesity (*p* = 0.01) (median ± range, *n* = 6). *Middle:* MSCs were incubated with DHE after incubation with or without Antimycin A (10 μM) to assess O_2_^•‐^. Two‐way ANOVA: Effect of Antimycin A (*p* < 0.0001) and obesity (*p* = 0.9) (median ± range, *n* = 6). *Bottom*: MSCs were incubated with mitoSOX after incubation with or without Antimycin A (10 μM) to assess mitochondrial O_2_^•‐^. Two‐way ANOVA: Effect of Antimycin A (*p* = 0.002) and obesity (*p* = 0.4) (median ± range, *n* = 6). For all graphs, Tukey's range post hoc test comparisons vs basal condition: # *p* < 0.05; ###*p* < 0.001; ####**p < **0.0001). (B) SOD1/2, GPX1 and CAT gene expression were measured for basal and H₂O₂‐induced conditions (250 μM) (median ± range, *n* = 7 NW‐MSCs and 7 OB‐MSCs). Two‐way ANOVA: Effect of BMI (SOD1: *p* = 0.001; SOD2: *p* < 0.0001; GPX1: *p* < 0.0001; CAT: *p* = 0.003. *p < 0.05 Tukey's range post hoc test NW‐MSCs versus OB‐MSCs. ^#^ *p* < 0.05 Tukey´s range post hoc test basal vs induced.

To assess intracellular ROS and antioxidant response, cells were exposed to H₂O₂ (400 μM) or tBHP (100 μM). Both treatments significantly increased ROS levels (H₂O₂: *p* = 0.0001; tBHP: *p* < 0.0001), with an additional effect of maternal obesity (*p* = 0.01). Moreover, H₂O₂ treatment increased intracellular ROS in NW‐MSCs (*p* = 0.001), but not in OB‐MSCs (*p* = 0.1). In contrast, tBHP treatment increased intracellular ROS in both NW‐MSCs and OB‐MSCs (*p* = 0.0004 and 0.01, respectively). Notably, intracellular ROS levels following H₂O₂ exposure were lower in OB‐MSCs compared to NW‐MSCs (*p* = 0.03, first row, Figure 3A).

### 2.7 NW‐MSCs and OB‐MSCs Increase Intracellular and Mitochondrial O_2_^•‐^ Production in Response to a Pro‐Oxidant Challenge

Following antimycin A treatment, total intracellular O_2_^•‐^ levels were increased in both groups. The results show an effect of antimycin A treatment (*p* < 0.0001), but not of maternal obesity (*p* = 0.9). Additionally, there was a significantly increased O_2_^•‐^ after treatment, compared to untreated cells for both groups (*p* < 0.0001 NW‐MSCs and OB‐MSCs, second row, Figure 3A). Further, mitochondrial O_2_^•‐^ levels were also assessed following antimycin A treatment. Antimycin A significantly increased mitochondrial O_2_^•‐^ production (*p* = 0.002), whereas no effect of maternal obesity was observed (*p* = 0.4). An increase in O_2_^•‐^ levels was detected in OB‐MSCs following antimycin A treatment compared with untreated cells (*p* = 0.04), while no difference was observed in NW–MSCs (*p* = 0.1, third row, Figure 3A).

### 2.8 Induced Antioxidant Gene Expression is Decreased in OB‐MSCs Compared to NW‐MSCs

There was an effect of maternal obesity over antioxidant gene expression levels in response to a challenge with H₂O₂ (SOD1: *p* = 0.001; SOD2: *p* < 0.0001; GPX1: *p* < 0.0001; CAT: *p* = 0.003). Further, SOD1, SOD2, and GPX1 mRNA significantly increased compared to the untreated cells in NW‐MSCs (*p* = 0.04, 0.02 and 0.03, respectively). None of the enzyme's mRNA increased after the H₂O₂ challenge compared to untreated cells in OB‐MSCs (Figure 3B). CAT gene expression was unaffected by the H₂O₂ challenge in both groups.

### 2.9 Protein Expression of FOXO1, Acetyl‐FOXO1, and SIRT2 in MSCs in Basal State

**Figure 4:**
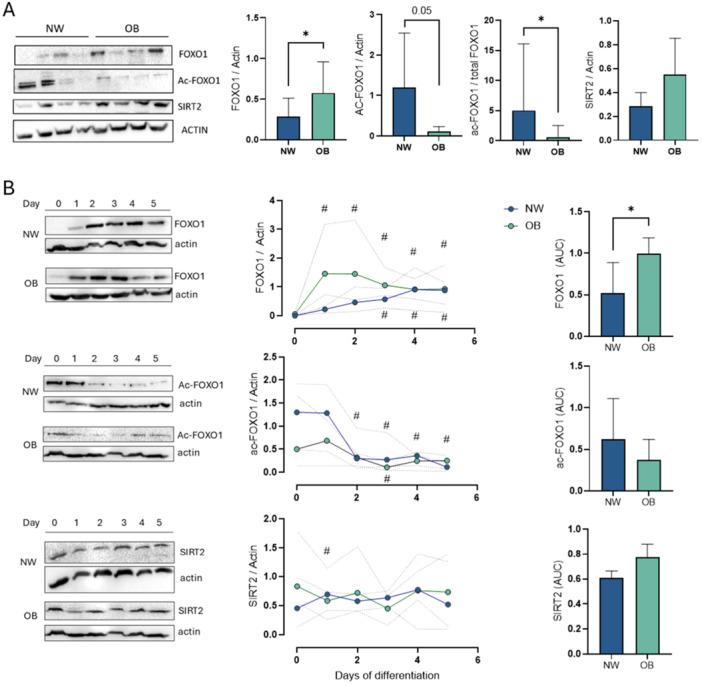
Early adipogenic expression of FOXO1, acetyl‐FOXO1 and SIRT2 in NW‐MSCs and OB‐MSCs. (A) FOXO1, acetyl‐FOXO1, ratio of acetyl‐FOXO1/total FOXO1 and SIRT2 on Day 0. *p < 0.05, Mann−Whitney *U* test (median ± range, *n* = 8 donors NW‐MSCs and 8 donors OB‐MSCs). (B) MSCs were cultured with adipogenic induction media for 0, 1, 2, 3, 4 or 5 days for protein expression of FOXO1, acetyl‐FOXO1, and SIRT2. Left: Representative western blots of NW‐MSCs and OB‐MSCs. For OB‐MSCs, acetyl‐FOXO1 and SIRT2 were probe with the same membrane and used the same actin as loading reference. Center: Levels of proteins over time in NW‐MSCs and OB‐MSCs. Right: Area under the curve (AUC) of levels of proteins in NW‐MSCs vs OB‐MSCs (median ± range, *n* = 4 donors NW‐MSCs and 4 donors OB‐MSCs). ^#^ *p* < 0.05 Paired Wilcoxon signed‐rank test compared to Day 0 from the same group; *p < 0.05 Mann−Whitney *U* test AUC.

Protein expression was assessed in MSCs at Day 0 (basal state). FOXO1 expression was significantly higher in OB‐MSCs compared to NW‐MSCs (*p* = 0.03; Figure 4A). In contrast, levels of acetyl‐FOXO1 and acetyl‐FOXO1/total FOXO1 ratio were significantly decreased in OB‐MSCs compared to NW‐MSCs (*p* = 0.05 and *p* = 0.02, respectively). SIRT2 levels showed no difference between OB‐MSCs and NW‐MSCs (*p* = 0.08).

### 2.10 Protein Expression of FOXO1, Acetyl‐FOXO1, and SIRT2 in MSCs During Early Adipogenesis

We next assessed the expression of these proteins in the first 5 days of adipogenic induction in MSCs. FOXO1 levels increased from Day 1 until Day 5 relative to Day 0 in OB‐MSCs (*p* < 0.05), whereas in NW‐MSCs a significant increase was observed only on Day 3 (*p* < 0.05, first row, Figure 4B). Consistently, FOXO1 area under the curve (AUC) was higher in OB‐MSCs compared to NW‐MSCs (*p* = 0.04, Figure 4B). Acetyl‐FOXO1 levels decreased from Day 2 onward in NW‐MSCs (Day 2 vs. 0: *p* = 0.04; Days 3–4: *p* < 0.05), whereas in OB‐MSCs a significant decrease was observed only at Day 3 (*p* < 0.05). Total acetyl‐FOXO1 AUC was not different between both groups (second row, Figure 4B). SIRT2 levels decreased on Day 1 in OB‐MSCs (*p* = 0.02) but not in NW‐MSCs (*p* = 0.5). No difference in SIRT2 levels over time or in AUC were observed between groups (third row, Figure 4B).

### 2.11 The Effect of ROS on FOXO1, Acetylation of FOXO1 and SIRT2 Protein Expression in MSCs During Early Adipogenesis

**Figure 5:**
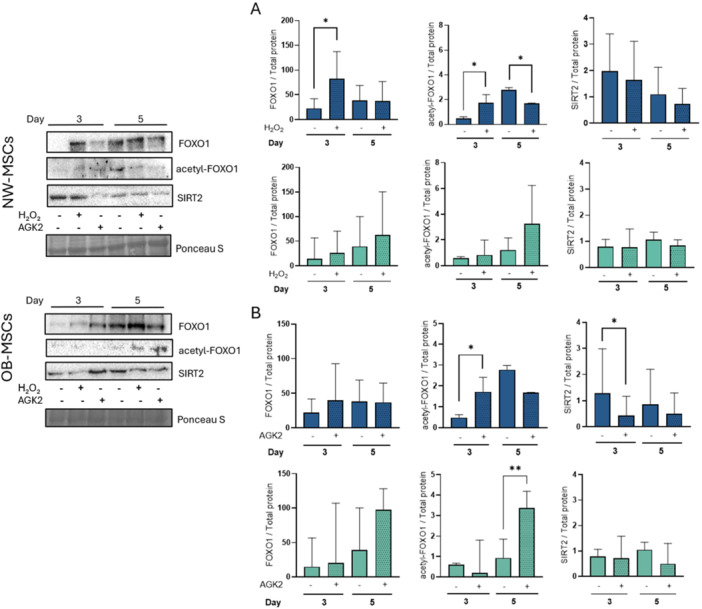
Induction of adipogenesis in NW‐MSCs and OB‐MSCs in the presence of H₂O₂ or AGK2. Cells were cultured with adipogenic induction media for 3 and 5 days, to assess the effect of (A) H₂O₂ (250 μM) or (B) AGK2 (10 μM), a SIRT2 inhibitor, on FOXO1, acetyl‐FOXO1 and SIRT2 levels. NW‐MSCs (blue), OB‐MSCs (green) (median ± range, *n* = 4 donors NW‐MSCs and 4 donors OB‐MSCs). *p < 0.05, Paired Wilcoxon signed‐rank test. Representative Western blots are shown in the left side.

To determine whether activation of the FOXO1‐SIRT2 pathway during adipogenesis is ROS‐dependent, adipogenic induction was performed in the presence of H₂O₂ (250 uM) as a mild oxidative challenge. Consistent with previous results, FOXO1 expression in NW‐MSCs increased at Days 3 and 5 of adipogenesis. H₂O₂ further increases FOXO1 expression at Day 3 (*p* = 0.01), but not at Day 5, compared to untreated cells (Figure 5A). In OB‐MSCs, H₂O₂ had no significant effect on FOXO1 expression at either time point (*p* = 0.4 and *p* = 0.2. In NW‐MSCs, H₂O₂ induced significantly higher levels of acetyl‐FOXO1 at Day 3 (*p* = 0.01), but decreased at Day 5 (*p* = 0.01, Figure 5A). In contrast, OB‐MSCs showed no effect of H₂O₂ at Day 3, with a trend toward increased acetyl‐FOXO1 at Day 5 following H₂O₂ stimulation (*p* = 0.06). SIRT2 levels were not affected by H₂O₂ treatment during adipogenesis in either NW‐MSCs or OB‐MSCs (Figure 5A).

### 2.12 The Effect of SIRT2 on FOXO1 and Acetyl‐FOXO1 in NW‐MSCs and OB‐MSCs During Early Adipogenesis

Next, we evaluated whether inhibition of SIRT2 affects the expression of FOXO1 and acetyl‐FOXO1. SIRT2 was inhibited using AGK2, a selective SIRT2 inhibitor (Yu et al. 2018) (Supporting Information S1: Figure 1). In NW‐MSCs, treatment with AKG2 significantly reduced SIRT2 protein expression at day 3 of adipogenesis (*p* = 0.04). This effect was not seen in OB‐MSCs (Figure 5B). Further, AGK2 treatment did not affect FOXO1 expression in either NW‐MSCs or OB‐MSCs (Figure 5B). However, in NW‐MSCs, AGK2 increased acetyl‐FOXO1 levels on day 3, compared to untreated cells (*p* = 0.03). By Day 5, there was a trend to lower acetyl‐FOXO1 levels in the presence of AGK2 (*p* = 0.06). In OB‐MSCs, acetyl‐FOXO1 was higher at day 5 compared to untreated cells (*p* = 0.006).

### 2.13 Subcellular Localization of acetyl‐FOXO1 in OB‐MSCs and NW‐MSCs

**Figure 6:**
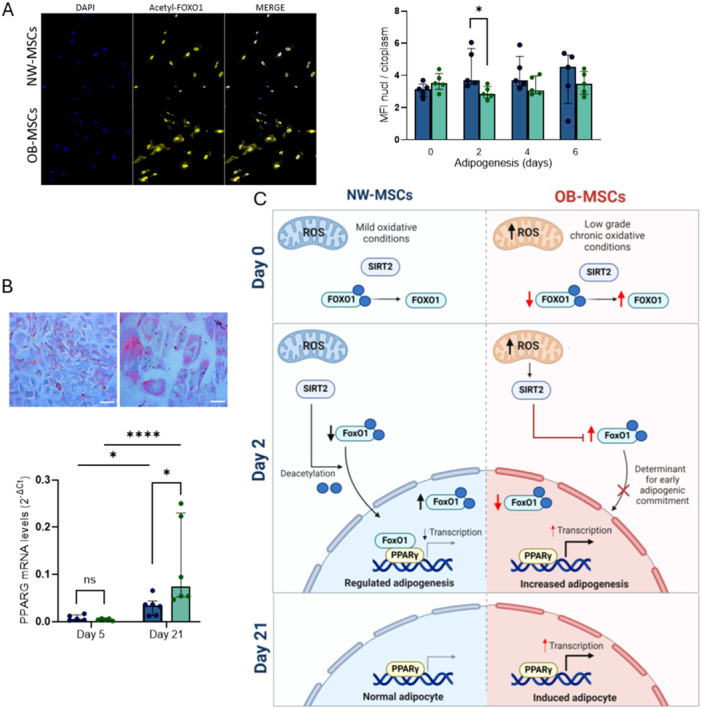
Subcellular localization of acetyl‐FOXO1 and adipogenesis in MSCs. (A) NW‐MSCs and OB‐MSCs were cultured with adipogenic induction medium and stained for acetyl‐FOXO1 during adipogenesis. Mean fluorescence intensity (MFI) of nuclear‐cytoplasmatic ratio was calculated for localization. Representative images correspond to Day 2 for NW‐MSCs (top) and OB‐MSCs (bottom) (median ± range, *n* = 6 donors NW‐MSCs and 6 donors OB‐MSCs). *p < 0.05, Mann−Whitney *U* test. (B) Lipid staining by Oil Red O in NW‐MSCs on day 21 (scale bar: 100 μM left; 10 μM right). PPARγ gene expression (*PPARG*) at Day 5 and 21 of adipogenesis in NW‐MSCs and OB‐MSCs (Median ± interquartile range, *n* = 6 donors NW‐MSCs and 6 donors OB‐MSCs). Effect of day over PPARγ (*p* < 0.0001, two‐way ANOVA); effect of obesity over PPARγ (*p* = 0.2, two‐way ANOVA). *p < 0.05 Tukey's range post hoc test, ****p < 0.0001 Tukey's range post hoc test. (C) Proposed mechanism of increased adipogenesis in OB‐MSCs compared to NW‐MSCs: MSCs of neonates from mothers with obesity have higher levels of total FOXO1 and SIRT2, together with lower acetyl‐FOXO1, which induces an early commitment in response to adipogenic induction. On early Day 2 of adipogenesis, acetyl‐FOXO1 localizes mainly in the cytoplasma for OB‐MSCs, therefore releases PPARγ for adipogenic gene transcription. Finally, on Day 21, in vitro adipocytes of OB‐MSCs show higher levels of PPARG gene expression, compared to NW‐MSCs. Created in BioRender.

To further investigate the role of acetyl‐FOXO1 during adipogenesis, subcellular localization was assessed on Days 0, 2, 4, and 6 of adipogenesis to evaluate its trafficking during early adipogenesis in both groups. At Day 0, there was no difference in the nuclear/cytoplasmatic ratio of acetyl‐FOXO1 between NW‐MSCs and OB‐MSCs, indicating similar localization in both groups at baseline. Nevertheless, at Day 2, the nuclear/cytoplasmatic ratio of acetyl‐FOXO1 was lower in OB‐MSCs compared with NW‐MSCs (*p* = 0.04, Figure 6A). At Days 4 and 6, no differences in the nuclear/cytoplasmatic ratio of acetyl‐FOXO1 were observed between both groups.

### 2.14 PPARγ Induction During Adipogenesis in NW‐MSCs and OB‐MSCs

PPARγ is a key regulator of the adipogenic transcriptional machinery (Lefterova et al. 2014). In our model, adipogenic commitment was confirmed by the presence of lipid droplets positive for Oil red O staining at Day 21 (Figure 6B) and the presence of both isoforms, PPARγ1 and PPARγ2 (Supporting Information S1: Figure 2). To determine whether obesity affects PPARγ, we evaluated PPARγ gene expression at Day 5 (early adipogenesis) and Day 21 (mature adipocytes). Two‐way ANOVA revealed a significant effect of the day of adipogenesis on PPARγ expression (*p* = 0.0001), whereas obesity had no overall effect (*p* = 0.2, Figure 5B). Post hoc analysis showed increased PPARγ expression on Day 21 compared to Day 5 of adipogenesis for both groups (NW‐MSCs: *p* = 0.001; OB‐MSCs: *p* = 0.0001). At Day 5, no differences in PPARγ expression were observed between NW‐MSCs and OB‐MSCs (*p* = 0.7). On Day 21 of adipogenesis, OB‐MSCs exhibited higher PPARγ expression compared to NW‐MSCs (*p* = 0.03).

## 3 Discussion

This study evaluated stemness and redox balance in adipocyte progenitor cells, the MSCs, derived from neonates of women with NW and with obesity. OB‐MSCs exhibited reduced self‐renewal, clonogenic capacity, and initial growth rate, along with higher mitochondrial ROS and evidence of redox adaptations. We further investigated the adipogenic pathway involving SIRT2, FOXO1, and PPARγ protein activation during early adipogenesis and under oxidative stress conditions. OB‐MSCs displayed higher FOXO1, reduced acetyl‐FOXO1 levels, and increased cytoplasmatic localization, a pattern consistent with reduced FOXO1‐mediated repression of PPARγ and earlier adipogenic commitment. As FOXO1 dynamics were modulated by H₂O₂, we propose that oxidative stress associated with maternal obesity may influence early adipogenic pathways in neonates from mothers with obesity (Figure 6C). These findings suggest that adipocyte precursor cells may already be metabolically programmed at birth in the offspring of mothers with obesity.

### 3.1 MSCs Stemness and Redox Balance

OB‐MSCs showed lower expression of the pluripotency marker OCT3/4 compared with NW‐MSCs. This indicates diminished pluripotency and self‐renewal capacity (Rosner et al. 1990; Niwa et al. 2000), which was further reflected by the reduced clonogenic capacity of OB‐MSCs upon passaging. Such characteristics are commonly associated with an aging‐like phenotype in stem cells (Fong et al. 2011; Di Micco et al. 2021). Previous studies have highlighted the role of cellular aging and senescence signaling in impairing adipogenesis and contributing to metabolic dysfunction (Chen et al. 2016; Narasimhan et al. 2022; Smirnova et al. 2023). Therefore, our findings suggest that OB‐MSCs may exhibit features of cellular aging, and future studies should further investigate aging‐ and senescence‐related markers.

In addition, OB‐MSCs exhibited higher mitochondrial O_2_^•‐^ levels compared to NW‐MSCs, demonstrating mitochondrial stress. These findings are consistent with those of Iaffaldano et al. who reported lower oxygen consumption rates and a reduced mitochondrial content in OB‐MSCs compared to NW‐MSCs (Iaffaldano et al. 2018). A lower oxygen consumption rate is known to increase electron leakage under various energetic conditions, which, in turn, leads to elevated mitochondrial O_2_^•‐^ production (Murphy 2009; Liesa and Shirihai 2013). Furthermore, Baker et al. characterized mitochondrial gene expression in OB‐MSCs and noted significant changes during adipogenic differentiation, including upregulation of the mitochondrial respiratory chain genes, alongside downregulation of mitochondrial biogenesis and mitophagy pathways (Baker et al. 2017). Together, these findings support the presence of mitochondrial dysfunction and oxidative stress in OB‐MSCs.

SOD2 was downregulated in OB‐MSCs compared to NW‐MSCs under basal conditions, denoting reduced mitochondrial antioxidant capacity. SOD2, a manganese‐dependent SOD (MnSOD), is the primary enzyme responsible for scavenging O_2_^•‐^ in the inner mitochondrial matrix and represents the first line of defense against mitochondrial oxidative damage (Hewitt and Degnan 2023). Reduced basal SOD2 levels are consistent with the elevated mitochondrial O_2_^•‐^ observed in OB‐MSCs and further support the presence of mitochondrial dysfunction in these cells. Because mitochondrial function plays an important role in the pathophysiology of obesity‐associated metabolic disorders, our findings provide evidence that such alterations may already be present in neonatal progenitor cells, potentially contributing to increased metabolic disease risk later in life (Zamora‐Mendoza et al. 2018; Domingues et al. 2019; Garcia‐Irigoyen et al. 2021).

Our findings reveal elevated total GSH levels in OB‐MSCs compared to NW‐MSCs, likely representing a compensatory response to the impaired GPX1 response. Reduced GPX1 activity may limit GSH consumption, leading to its accumulation as a compensatory mechanism against oxidative stress (Schafer and Buettner 2001; Lubos et al. 2011; Brigelius‐Flohé and Maiorino 2013; Jiao et al. 2017). Similar alterations have been reported in individuals with obesity (Kobayashi et al. 2009; Alkazemi et al. 2021), and may reflect enhanced GSH synthesis to control cytosolic ROS levels. When exposed to H₂O₂, OB‐MSCs displayed attenuated intracellular H₂O₂ levels together with a blunted GPX1 response, suggesting altered GSH metabolism.

We assessed cytosolic antioxidant capacity by using prooxidant challenges. OB‐MSCs showed lower intracellular ROS levels following H₂O₂ exposure, compared to NW‐MSC, suggesting more efficient primary antioxidant defenses, potentially due to higher GSH availability. However, we do not discard the contribution of additional redox systems such as peroxiredoxins and thioredoxins (Sies et al. 2017). Under stronger oxidative stress induced by tBHP (Alía et al. 2005), no differences in intracellular ROS were observed between NW‐MSCs and OB‐MSCs. These results support an adaptive response to mild oxidative stress in OB‐MSCs.

Elevated oxidant levels can induce adaptive stress responses that enhance cellular resilience, a phenomenon known as hormesis or mitohormesis (Ristow and Schmeisser 2014; Vaiserman 2011). Such adaptive mechanisms can increase tolerance to oxidative stress, thereby preventing excessive strain on the system while promoting cellular longevity (Vaiserman 2011; Sies et al. 2022). These responses are particularly relevant during early developmental stages due to the plasticity of individuals (Vaiserman 2011; Ristow and Schmeisser 2014; Li et al. 2019). In our study, OB‐MSCs showed a blunted transcriptional response of antioxidant enzymes SOD1, SOD2, and GPX1 following oxidative challenge. Whether this attenuated response confers a protective adaptation or reflects impaired stress signaling remains to be determined.

### 3.2 MSCs and Adipogenesis

We found that OB‐MSCs exhibited higher basal FOXO1 expression and an earlier peak of FOXO1 expression during adipogenesis compared to NW‐MSCs. This suggests that OB‐MSCs may initiate adipogenic differentiation earlier than NW‐MSCs. These findings are in agreement with enhanced adipogenic commitment that has been previously reported in OB‐MSCs (Iaffaldano et al. 2013; Chen et al. 2016; Boyle et al. 2016). FOXO1 is a transcription factor that responds to oxidative stress by regulating the expression of antioxidant genes, including SODs, CAT, GPXs, thioredoxins and peroxiredoxins (Higuchi et al. 2013; Klotz et al. 2015; Weng et al. 2016; Qiu et al. 2019). Therefore, the increased FOXO1 expression observed in OB‐MSCs may represent a response to mitochondrial oxidative stress. Additionally, FOXO1 is induced by adipogenic stimuli during early adipogenesis to regulate (1) cell cycle by stimulating growth arrest by the upregulation of p21 and p27 to inhibit clonal expansion (Adachi et al. 2007), and (2) indirect activation of CEBP/s during adipogenesis (Hishida et al. 2009). Because FOXO1 expression occurred earlier in OB‐MSCs, these mechanisms may contribute to accelerated adipogenic progression in these cells.

Exposure to H₂O₂ increased FOXO1 expression in NW‐MSCs; however, FOXO1 expression in OB‐MSCs was not responsive to H₂O₂. FOXO1 has been described to regulate pathways widely associated with stress adaptation to confer resilience and ultimately promote lifespan (Klotz et al. 2015; Rodriguez‐Colman et al. 2024). Considering the mitochondrial oxidative stress and adaptive redox adaptations observed in OB‐MSCs, it is possible that the obesogenic intrauterine environment influences FOXO1‐mediated stress tolerance mechanisms. Future studies should explore additional FOXO1‐regulated pathways potentially affected by maternal obesity, including gluconeogenesis, the insulin pathway and cell cycle regulation (Klotz et al. 2015).

Our results further showed lower acetyl‐FOXO1 levels and a reduced acetyl‐FOXO1/total FOXO1 ratio in OB‐MSCs compared to NW‐MSCs at day 0. This suggests that FOXO1 is predominantly deacetylated and likely transcriptionally active (Chen et al. 2019; Nakae et al. 2003; Zou et al. 2014; Zhang et al. 2022). By day 2, OB‐MSCs exhibited a lower nuclear/cytoplasmatic acetyl‐FOXO1 ratio, indicating increased cytoplasmatic localization. Because acetylation regulates both FOXO1 DNA binding and nucleo‐cytoplasmatic shuttling (Daitoku et al. 2004; Matsuzaki et al. 2005), cytoplasmatic retention may limit its ability to repress PPARγ transcription. Reduced repression of PPARγ could facilitate adipogenic differentiation in OB‐MSCs (Nakae et al. 2003; Armoni et al. 2006; Jing et al. 2007; Nakae et al. 2008; Wang and Tong 2009). After day 2, decreasing acetyl‐FOXO1 levels in both groups coincided with cell cycle arrest and the onset of adipogenesis, with earlier PPARγ activation likely occurring in OB‐MSCs. Nevertheless, phosphorylation of FOXO1 also plays an important regulatory role, and future studies should investigate FOXO1 phosphorylation dynamics during adipogenesis (Zou et al. 2014).

SIRT2 is a NAD+ dependent deacetylase that regulate multiple cellular processes, including oxidative status (Madsen et al. 2016). Although H₂O₂ exposure did not alter SIRT2 protein levels, acetyl‐FOXO1 levels changed, suggesting that H₂O₂ may modulate SIRT2 activity rather than its expression. We did not examine the participation of other mechanisms regulating acetylation/deacetylation of FOXO1, which could also be sensitive to H₂O₂ to attribute this effect (Chen et al. 2019). SIRT2 was significantly inhibited by AGK2 in NW‐MSC at Day 3 of adipogenesis, with a weaker effect at Day 5, whereas no effect was observed in OB‐MSCs. This may indicate increased robustness of SIRT2 in OB‐MSCs, potentially as an adaptation to higher ROS levels. Moreover, because acetyl‐FOXO1 levels were increased on Day 5 of adipogenesis in OB‐MSCs following AGK2 treatment, we suggest the involvement of additional regulatory mechanisms, such as CBP/p300 acetylation or SIRT1 deacetylase activity in adipogenesis. Future studies should consider evaluating these other important mechanisms that regulate acetyl‐FOXO1 in OB‐MSCs (Xie et al. 2012), while also knockdown and rescue experiments could provide valuable insights into FOXO1 dynamics (Jing et al. 2007).

Because FOXO1 dynamics differed between NW‐MSCs and OB‐MSCs during early adipogenesis, we hypothesized that PPARγ expression would also differ between groups. Although OB‐MSCs displayed reduced nuclear acetyl‐FOXO1 localization at Day 2, which strongly suggests less repression of FOXO1 on PPARγ (Armoni et al. 2006), we found no difference in PPARγ expression on Day 5. Our data suggest that SIRT2‐FOXO1 induction functions as an early molecular gatekeeper that determines the permissiveness for activation of PPARγ transcription. This likely reflects the early stage of adipogenic differentiation at this time point, as robust PPARγ transcription typically occurs after Day 6 and is regulated by multiple coordinated mechanisms, including insulin signaling, C/EBPs transcription factors, and chromatin remodeling (Dominici et al. 2006; Chen et al. 2019). Direct evaluation of FOXO1–PPARγ binding activity would provide further mechanistic insight. Nevertheless, by Day 21, PPARγ expression was significantly higher in OB‐MSCs compared to NW‐MSCs, confirming enhanced adipogenic commitment, as seen in other studies (Iaffaldano et al. 2013; Boyle et al. 2016; Chen et al. 2016).

Finally, donor variability represents an inherent limitation when using primary human MSCs, particularly across BMI categories. Although this variability may increase experimental heterogeneity, it also reflects physiological conditions and enhances translational relevance, as consistent differences between normal‐weight and obese donors were still observed. Another limitation of this study is the lack of detailed maternal and neonatal clinical data. Future studies integrating clinical data with molecular pathways will be essential to determine how maternal metabolic status programs adipocyte progenitor function and metabolic risk in the progeny.

## 4 Conclusions

In conclusion, maternal obesity impacts stemness, redox balance, and FOXO1 dynamics in neonatal MSCs, leading to dysregulated FOXO1 compared to NW‐MSCs. These findings support a model in which maternal obesity induces mitochondrial oxidative stress in neonatal MSCs, disrupting FOXO1 regulation, thereby shifting early adipogenic signaling toward enhanced adipocyte commitment. This mechanism may expand the adipocyte precursor pool, potentially predisposing the progeny to metabolic disorders later in life.

## 5 Methods

### 5.1 Experimental Design

MSCs were isolated from Wharton's jelly of umbilical cords from neonates of mothers with normal weight and mothers with obesity. Basal MSCs were analysed for stemness properties and redox parameters. Cells were exposed to 250 μM H₂O₂ to assess antioxidant enzyme responses, and to 400 μM H₂O₂, 100 μM tert‐butyl hydroperoxide (tBHP), or 20 μM Antimycin A, to measure intracellular ROS and O_2_^•‐^ production in response to oxidative stress. Then, MSCs were subjected to adipogenesis to evaluate SIRT2, FOXO1 expression, and ROS responses (Day 0–5). FOXO1 involvement was confirmed by acetyl‐FOXO1 localization, and adipogenesis was verified by PPARγ expression.

### 5.2 Subjects

Umbilical cords were anonymously obtained from the placentas of women with obesity or normal weight, who delivered at the University Medical Centrum Groningen (UMCG) maternity ward, Groningen, The Netherlands, from August 2021 to February 2023. Umbilical cords were obtained from biological waste material following routine childbirth procedures. No specific ethical permission was required as tissue was classified as medical waste and was de‐identified, with no donor information collected. The study complied with all relevant institutional and national ethical regulations regarding the use of biological materials. All procedures were also conducted according to the Helsinki Declaration. A pregestational maternal body mass index (BMI) > 30 kg/m² was considered for the group of women with obesity (OB, *n* = 15) and 18.5–24.5 kg/m² for women with normal weight (NW, *n* = 15). The inclusion criteria included women > 18 years, single, and term pregnancies (>37 weeks). The exclusion criteria included women with gestational diabetes, preeclampsia, preterm birth, and neonatal complications. All characteristics of the mother and newborn were blinded, apart from the woman's pregestational BMI (weight, height) and gestational weight.

### 5.3 Isolation of Wharton's Jelly‐Derived MSCs

Umbilical cords were obtained from deliveries and immediately processed to get MSCs primary cultures. MSCs were isolated using the explant method (La Rocca et al. 2009). Briefly, the umbilical cord was cut into three cm pieces, and the blood vessels were discarded. Wharton's jelly explants were plated and cultured with Dulbecco's modified Eagle medium containing 10 mM glucose (DMEM, Gibco, Thermo Fischer Scientific), 10% Fetal calf serum (FCS, Sigma‐Aldrich), 5.000 UI/mL Penicillin‐Streptomycin (Gibco, Thermo Fischer Scientific), and maintained at 37°C in 5% CO₂. Media was changed every 4 days, and culture was maintained for 10–14 days. By this time, a solid population of cells had sprouted out from the explants and had covered the explant perimeter. Subsequently, the sprouted cells were trypsinized (Gibco, Thermo Fischer Scientific) and expanded into further passages by seeding at 4.000/cm² in culture flasks. The medium was changed every 3 days, and cells were maintained in the same culturing conditions. Cells were passaged after 6 days of culture (passages 1–3). Unless otherwise stated, all experiments were performed in fresh cells on passages 2–3.

### 5.4 Immunophenotyping

Surface markers were characterized according to the International Society of Cell Therapy criteria (Dominici et al. 2006) Briefly, cells were detached with trypsin and centrifuged at 2500*g* for 3 min. After this, 1 × 10^6^ cells were resuspended in 100 μL of FACS buffer (2% FCS in DPBS) in FACS tubes (Falcon, Corning) and incubated for 30 min in the dark with antibodies for CD73 (APC, 1:50, Miltenyi Biotec), CD90 (PE, 1:50, Miltenyi Biotec), CD105 (BV421, 1:20, BioLegend), CD34 (PE‐Dazzle 594, 1:20, Biolegend), CD45 (BV785, 1:20, BioLegend), and CD11b (APC‐Vio770, 1:50, Miltenyi Biotec). Then, cells were washed with 2 ml FACS buffer and centrifuged at 2250*g* for 3 min, twice. Finally, cells were resuspended in 300 μL FACS buffer. Samples were measured by Novocyte Quanteon II Flow Cytometer (Agilent), and data were analysed in NovoExpress (Agilent). Events were gated for side scatter height (SSC‐H) vs forward scatter height (FSC‐H) to exclude debris and FSC‐H versus forward scatter area (FSC‐A) to exclude doublets. Further, populations were gated in the fully stained samples, using gate settings in the unstained samples.

### 5.5 Lineage Commitment

Cells were seeded in a 24‐well plate at 10,000 cells/cm². When cells were 80% confluent, they were incubated with differentiation media for each lineage using the following induction media: (a) adipogenic induction medium: DMEM (25 mM glucose; Gibco, Thermo Fischer Scientific) supplemented with 10% FCS (Sigma‐Aldrich), 1 nM insulin (Lonza Bioscience), 0.5 mM 3‐isobutyl‐1‐methylxanthine (Sigma‐Aldrich), 0.1 μM dexamethasone (Sigma‐Aldrich); (b) osteogenic induction medium: DMEM (25 mM glucose, Gibco, Thermo Fischer Scientific) supplemented with 10% FCS (Sigma‐Aldrich), 10 mM β‐glycerophosphate (Sigma‐Aldrich), 0.1 μM dexamethasone (Sigma‐Aldrich), 0.05 mM ascorbic acid (Sigma‐Aldrich); and (c) myogenic induction medium: DMEM (25 mM glucose, Gibco, Thermo Fischer Scientific) supplemented with 10% Fetal calf serum (FCS, Sigma‐Aldrich), 0.1% TGF‐β1 (PeproTech). The medium was changed every 3 days, and differentiation was carried out through 14 (osteogenic/myogenic/adipogenic) and 21 days (adipogenic). After this time, cells were fixed in the plate with 2% PFA (Sigma‐Aldrich). Experiments were performed in duplicates.

### 5.6 Staining of Lineage Commitment

For adipocyte staining, fixed cells in the plate were washed with water, rinsed with 60% 2‐propanol, and immediately stained with Oil red O (Sigma‐Aldrich) for 30 min. Nuclei were briefly stained with hematoxylin (Sigma‐Aldrich). For osteoblast staining, fixed cells were washed with DPBS and stained in 0.5% Alizarin Red (Sigma‐Aldrich) for 10 min. Nuclei were stained with hematoxylin. Images were acquired by a Leica DM IL Led microscope (Leica Microsystems). For smooth muscle staining, fixed cells were washed with DPBS and incubated with Phalloidin‐AlexaFluor488 (1:250; Thermo Fischer Scientific) and DAPI (1:5000, Roche Diagnostics) for 30 min. Images were acquired with EVOS FL (Life Technologies, Fischer Scientific).

### 5.7 SSEA‐4 Labelling

Passage 2 cells were seeded at 2000 cells/cm² in Lab‐Tek chambers (Thermo Fischer Scientific) and fixed with 2% PFA (Sigma‐Aldrich). After DPBS (Gibco, Thermo Fischer Scientific) washes, cells were incubated with SSEA‐4 (1:500, Invitrogen) for 2 h, followed by anti‐mouse Alexa Fluor 546 (Thermo Fischer Scientific) for 1 h. Cells were further stained with DAPI (Roche Diagnostics) for 15 min. Images were acquired using EVOS FL (Life Technologies, Fischer Scientific). Fluorescence intensity was quantified with Image J (NIH) as: [Fluorescent intensity = mean cell intensity—mean background intensity]. Background fluorescence was measured from each cell area (Hartig 2013).

### 5.8 RNA Isolation and cDNA Synthesis

Cells were cultured in 6‐well plates at 5000 cells/cm². For the induced state, we used an oxidative challenge with 250 μM H₂O₂ (Merck) for 6 h. Pilot experiments indicated that this challenge induced antioxidant gene expression without affecting cell viability (Supporting Information S1: Figure 3). After reaching confluency, cell lysates were collected with TRIzol (Invitrogen). Total RNA was isolated as described by the manufacturer (Chomczynski 1993). Pellet was reconstituted in water, and RNA concentration and purity were measured with a NanoDrop ND‐100 UV‐Vis spectrophotometer (NanoDrop Technologies). A total of 500 ng of RNA was used for reverse transcriptase cDNA synthesis following the standard protocol for RevertAid Reverse Transcriptase (Thermo Fischer Scientific Inc.).

### 5.9 OCT3/4, Antioxidant Enzymes and PPARγ Gene Expression

Quantitative PCR (qPCR) was performed with TaqMan Assay for OCT 3/4 (POU5F1 Hs04260367_gH, Thermo Fischer Scientific) according to the manufacturer's instructions. Glyceraldehyde 3‐phosphate dehydrogenase (GAPDH Hs02786624_g1, Thermo Fischer Scientific) was used as the housekeeping gene. For antioxidant enzymes and PPARγ, qPCR was performed with FastStart Universal SYBR Green Master (Roche Diagnostics) for SOD1, SOD2, GPX1, CAT, and PPARG according to the manufacturer's instructions. β‐2‐Microglobulin (B2M), GAPDH and 60S ribosomal protein P0 (RPLP0) were used as housekeeping genes. qPCR was performed in Viia7 Real‐time PCR System (Thermo Fischer Scientific). The threshold cycle (*C*_t_) for gene amplification was detected and normalized as a relative expression using 2ˆdelta *C*_t_ (Δ*C*_t_ = *C*_t_ [gene of interest]—*C*_t_ [average housekeeping genes]) (Supporting Table S2—primers list).

### 5.10 Colony Forming Unit Assay

Cells were seeded at low density (10 and 100 cells/cm²) for passage 2 and passage 5. The media was changed every 3 days. After 14 days, cells were fixed in 2% PFA (Sigma‐Aldrich) and stained with 0,05% Crystal Violet (Sigma‐Aldrich). Images were acquired by Leica DM IL Led microscope (Leica Microsystems). Colonies with more than 50 cells were considered positive colonies. Experiments were performed in duplicates.

#### 5.10.1 Growth Kinetics

MSCs were seeded on 12‐well plates at 2000 cells/cm². The medium was changed every 3 days for 10 days. Every 48 h, two arbitrary wells were trypsinized (Gibco, Thermo Fischer Scientific), and cells were counted in triplicate with a Coulter Counter Z2 (Beckman Coulter Diagnostics).

### 5.11 Total Intracellular ROS Levels

Basal and induced total intracellular ROS levels were assessed with 2′,7′‐dichlorodihydrofluorescein diacetate (H2DCFDA) (Thermo Fischer Scientific) (Murphy 2009). Briefly, cells were seeded at 3000/cm² in 24‐well plates and cultured until confluence. After reaching confluency, cells were detached with trypsin (Gibco, Thermo Fischer Scientific) and centrifuged at 1200*g* for 5 min. After this, cells from each well were resuspended in 1 mL of DPBS (Gibco, Thermo Fischer Scientific) in FACS tubes (Falcon, Corning), and incubated with H2DCFDA (5 μM) for 30 min at 37°C in the dark. For the prooxidant challenge, cells were incubated for the last 15 min with 400 μM of hydrogen peroxide (H₂O₂, Merck) as a physiological ROS, or with 100 μM of tBHP (Sigma‐Aldrich) as a stable non‐physiological ROS, at 37°C in the dark. Untreated cells were incubated at the same time. Cells were washed with 1 ml DPBS, centrifuged at 1200*g* for 5 min and resuspended in 300 μL DPBS. Then, 0.1 μM of ZombieNIR (BioLegend) was added to evaluate cell viability. Five thousand events were measured in the FITC channel in the Novocyte Quanteon II Flow Cytometer (Agilent). Events were gated for SSC‐H vs FSC‐H, and FSC‐H versus FSC‐A to exclude doublets and dead cells, respectively (Supporting Information S1: Figure 4). Data was analysed with NovoExpress (Agilent).

### 5.12 Total Intracellular and Mitochondrial O_2_^•‐^ Levels

O_2_^•‐^ levels were evaluated with dihydroethidium (DHE) and MitoSOX (Thermo Fischer Scientific) (Murphy 2009). Briefly, cells were seeded at 3.000/cm² for each condition in 24‐well plates and cultured until confluence. After reaching confluency, cells were detached with trypsin (Gibco, Thermo Fischer Scientific) and centrifuged at 1200*g* for 5 min. After this, cells were resuspended in 1 ml of DMEM (Gibco, Thermo Fischer Scientific) without FBS, in FACS tubes (Falcon, Corning) and incubated with DHE (5 μM) or mitoSOX (1 μM) for 30 min at 37°C in the dark. For the pro‐oxidative challenge, cells were incubated in suspension for the last 15 min with 10 μM of Antimycin A (Sigma‐Aldrich) at 37°C in the dark. Untreated cells, i.e. without pro‐oxidative challenge, were incubated at the same time. For each condition, cells were washed with 1 mL DPBS, centrifuged at 1200*g* for 5 min, and resuspended in 300 μL DPBS. Then, 0.1 μM of ZombieNIR (BioLegend) was added for cell viability assessment. Five thousand events were measured in the PE channel by Novocyte Quanteon II Flow Cytometer (Agilent). Events were gated for SSC‐H versus FSC‐H, and FSC‐H versus FSC‐A to exclude doublets, and dead cells were excluded. (Supporting Information S1: Figure 4). Data was analysed with NovoExpress (Agilent).

### 5.13 GSH Levels

Cells were seeded at 5000 cells/cm² in six‐well plates and cultured in standard conditions until confluence. After reaching confluency, cells were detached with trypsin (Gibco, Thermo Fischer Scientific) and centrifuged at 900*g* for 5 min. 1 × 10ˆ5 cells were resuspended in a buffer containing 50 mM sodium phosphate and 1 mM EDTA, sonicated, and stored at −80°C. After collecting all samples, they were thawed and deproteinized with 5% meta‐phosphoric acid (Sigma‐Aldrich). Oxidized (GSSG), reduced (GSH), and total GSH (GSHt) levels were measured with a GSH GSH/GSSG Assay kit (Sigma‐Aldrich), following the manufacturer's instructions. The optical density of samples was measured in a 96‐well plate reader (Biotek Epoch 2, Agilent) at 412 nm wavelength (time zero and 10 min). Data was calculated according to the standard curve. For GSSG, the process was performed in the presence of the scavenger 1‐methyl‐2‐vinylpyridinium triflate. Finally, total GSH levels were calculated with the following formula:

GSH μM = (GSHt)–[2 × (GSSG)].

### 5.14 Cell Culture and Adipogenic Induction

Cells were seeded at 7000 cells/cm² in six‐well plates and cultured with DMEM (Gibco, Thermo Fischer Scientific) (10 mM glucose), 10% FCS (Sigma‐Aldrich), Penicillin‐Streptomycin (Gibco, Thermo Fischer Scientific), and maintained at 37°C in 5% CO₂. When cells reached 80% confluency, adipogenesis was induced for 5 or 21 days with high glucose DMEM (25 mM glucose) supplemented with 10% FCS, 1% Penicillin‐Streptomycin (Gibco, Thermo Fischer Scientific), 1 nM insulin (1Lonza Bioscience), 0.1 μM dexamethasone (Sigma‐Aldrich), and 0.5 mM 3‐ isobutyl‐1‐methylxantine (Sigma‐Aldrich) (Scott et al. 2011). To simulate oxidative stress, during adipogenic induction, a mild oxidative challenge (250 μM H₂O₂, Merck) was added to the culture every 48 h. To inhibit SIRT2 expression, 10 μM AGK2 (Sigma‐Aldrich) was added to the culture medium every 48 h (Supporting Information S1: Figure 1).

### 5.15 Protein Collection and Western Blot Analysis

MSCs were lysed with RIPA buffer (Thermo Fischer Scientific) and Protease and Phosphatase Inhibitor Cocktail (Sigma‐Aldrich) on Day 0–5 of adipogenesis. 60 μg of proteins was run in 10% Sodium dodecyl‐sulfate polyacrylamide gel electrophoresis (SDS‐PAGE) for 1.5 h at 120 mV, transferred to a nitrocellulose membrane (Bio‐Rad) and blocked with 5% powdered non‐fat milk (Friesland Campina) or BSA (Sigma‐Aldrich) in ×1 Tris‐buffered saline plus 0.1% Tween T (TBST). Membranes were incubated overnight at 4°C with antibodies for FOXO1, acetyl‐FOXO1 Lys 294, SIRT2, PPARγ1 and 2, β‐actin, and α‐tubulin (Supporting Information S1: Table 1). Thereafter, membranes were washed and incubated with secondary antibodies conjugated with HRP for 1 h at room temperature (Supporting Information S1: Table 3). Only for experiments with ROS and AGK2, membranes were stained with Ponceau S for total protein as reference of protein quantity, because inhibition of SIRT2 can alter polymerization of actin (Min et al. 2018). Membranes were stripped and re‐probed for proteins of similar molecular weight (Restore, Thermo Fischer Scientific; Supporting Information S1: Figure 5). For OB‐MSCs samples, SIRT2 and acetyl‐FOXO1 were detected on the same membrane, which was subsequently stripped and reprobed for actin as a loading control; therefore, actin signals correspond to the same blot on days 0‐5. In contrast, for NW‐MSCs samples, proteins were analyzed on separate membranes. Membranes were washed with TBST, and signals were detected by chemiluminescence with SuperSignal West Pico PLUS Substrate (Thermo Fischer Scientific). Images were acquired with the ChemiDoc XRS+ Imaging System (Bio‐Rad). Data was quantified in ImageJ (NIH), and expressed relative to β‐actin, α‐tubulin, or total protein expression.

### 5.16 Localization of Acetyl‐FOXO1

Cells were seeded at 2000 cells/cm² in Labtek chambers (Thermo Fischer Scientific). After 48 h, in NW‐MSCs and OB‐MSCs adipogenesis was induced for 6 days as described above. At Day 0, 2, 4, and 6, cells were washed with DPBS and fixed with PFA 2% (Sigma‐Aldrich). Slides were washed with DPBS (Gibco, Thermo Fischer Scientific) and incubated with anti‐acetyl‐FOXO1 Lys 294 (Invitrogen, 1:500) for 2 h, washed again, and incubated with secondary anti‐rabbit Alexa Fluor 555 (Thermo Fischer Scientific, 1:500). Cells were further stained with DAPI (Roche Diagnostics) for 15 min. Images were taken by a Leica SP8 CLSM microscope (Leica Microsystems). For each image, Ilastik (Berg et al. 2019) was used to detect and separate the nuclear‐cytoplasmatic area. Further, Cell Profiler (Stirling et al. 2021) was used to quantify the mean fluorescent intensity of nuclear and cytoplasmatic area separately. Nuclear/cytoplasmatic ratio was calculated. Experiments were done in duplicate for each subject, and we analyzed 8–12 cells per field.

### 5.17 Statistical Analysis

The parameters investigated were expressed as the median value and 25th and 75th percentiles in Graphpad Prism (Graphpad Inc). Mann–Whitney *U* tests were used to compare between NW‐MSCs and OB‐MSCs. Data was log transformed, and Two‐way ANOVA and Tukey's post hoc test were used to assess the effect of passaging for clonogenic capacity assays (Passages 2 and 5), mRNA expression, ROS or O_2_^•‐^ levels, and time on PPARG gene expression, after transforming data to log2. *p* < 0.05 was considered statistically significant.

## Author Contributions

**Sofía Bellalta:** conceptualization, investigation, writing – original draft, methodology, visualization, writing – review and editing, software, formal analysis, project administration, data curation. **Erika Pinheiro‐Machado:** conceptualization, methodology, validation, writing – review and editing, formal analysis. **Theo Borghuis:** software, formal analysis, methodology. **Jelmer Prins:** conceptualization, validation, methodology, resources. **Torsten Plösch:** conceptualization, investigation, writing – review and editing, validation, project administration, data curation, supervision. **Paola Casanello:** conceptualization, funding acquisition, writing – review and editing, methodology, supervision. **Marijke Faas:** investigation, conceptualization, funding acquisition, writing – review and editing, writing – original draft, visualization, formal analysis, project administration, data curation, supervision.

## Ethics Statement

The authors assert that all procedures contributing to this work comply with the ethical standards of the relevant national guidelines on human experimentation and with the Helsinki Declaration of 1975, as revised in 2008, and have been approved by the institutional committees of the University Medical Center Groningen.

## Conflicts of Interest

The authors declare no conflicts of interest.

## Supporting information

Supporting File

## Data Availability

Data will be made available on request.
