# Supplementary material for: Maternal Obesity Programs Adipogenic Commitment in Neonatal Mesenchymal Stem Cells: A Link to Redox‐Dependent FOXO1 Signaling
Source: J Cell Physiol. 2026 Apr 28;241:e70178. doi: 10.1002/jcp.70178 (PMC13122739; doi:10.1002/jcp.70178)
Supplement: Supplementary file 1 — Supporting File [file JCP-241-0-s001.docx]

**Supplementary Material**

**Supplementary Table S1. Wharton’s jelly-derived MSCs exhibit MSCs immunophenotype**

| **Antigen** | **NW MSCs (%)** | **OB MSCs (%)** | **P value** |
| --- | --- | --- | --- |
| CD73 | 99.75 ± 0.1 | 99.77 ± 0.2 | 0.99 |
| CD90 | 97.53 ± 2.8 | 97.73 ± 3.0 | 0.83 |
| CD105 | 88.20 ± 4.0 | 89.42 ± 3.8 | 0.50 |
| CD34 | 0.18 ± 0.03 | 0.21 ± 0.1 | 0.99 |
| CD45 | 0.45 ± 0.3 | 0.65 ± 0.2 | 0.56 |
| CD11b | 0.28 ± 0.1 | 0.99 ± 0.7 | 0.30 |

P value of Mann-Whitney U test; n=3.

**Supplementary Table S2. Primers sequences and amplification conditions for RT‐qPCR**

| **mRNA**  **Target** | **Forward Sequence** | **Reverse Sequence** | **Efficiency (%)** |
| --- | --- | --- | --- |
| *SOD1* | GGTGTGGCCGATGTGTCTAT | CCTTTGCCCAAGTCATCTGC | 93 |
| *SOD2* | TGGGGTTGGCTTGGTTTCAA | TAGTAAGCGTGCTCCCACAC | 93 |
| *GPX1* | AGTGCGAGGTGAACGGTGCG | GGGGTCGGTCATAAGCGCGG | 94 |
| *CAT* | GTGCGGAGATTCAACACTGCCA | CGGCAATGTTCTCACACAGACG | 100 |
| *RPLP0* | AATCTCCAGGGGCACCATTG | GAACACCTGCTGGATGACCA | 100 |
| *GAPDH* | AGCCAACATCGTCAGACAC | GCCCAATACGACCAAATCC | 100 |
| *B2M* | GGTTTCATCCATCCATCCGACATT | ACGGCAGGCATACTCATCTT | 100 |
| *PPARG* | AACAGATCCAGTGGTTGCAG | AATTGCCATGAGGGAGTTGG | 94 |

**Supplementary Table S3. Antibodies used for protein quantification in Western Blot**

| **Antibody** | **Concentration** |
| --- | --- |
| FOXO1 - Invitrogen | 1:500 |
| acetyl-FOXO1 Lys 294 - Invitrogen | 1:1000 |
| SIRT2 - Cell Signaling | 1:500 |
| PPARγ - Cell Signaling | 1:250 |
| β-actin - Cell Signaling | 1:10000 |
| α-tubulin - Proteintech | 1:1000 |
| Rabbit anti-Mouse - Dako, Agilent | 1:5000 |
| Goat anti-Rabbit - Dako, Agilent | 1:5000 |


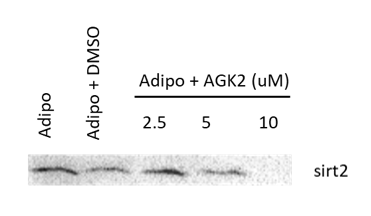

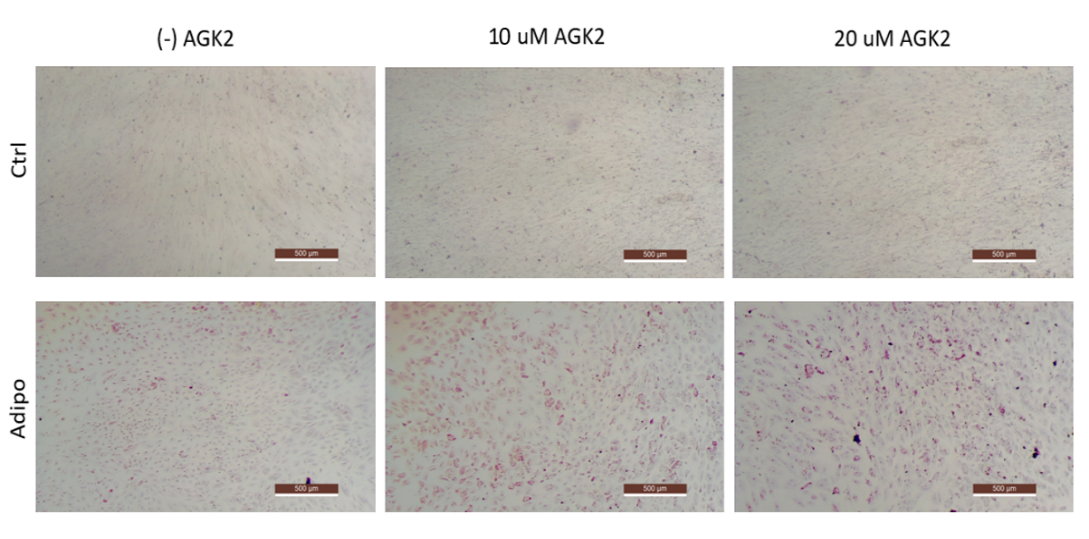


A

B

**Supplementary Figure S1. Inhibition of SIRT2 by AGK2 in NW-MSCs. NW-MSCs were cultured in the presence of AGK2 for inhibiton of SIRT2. A.** Protein expression of SIRT2 in NW-MSCs induced for 24 hours of adipogenesis in the presence of 2.5, 5 and 10 μM of AGK2, diluted in dimethyl sulfoxide (DMSO), indicating that 10 μM of AGK2 inhibits SIRT2 expression. **B**. NW-MSCs were induced for 21 days for adipogenesis in presence of 10 and 20 μM of AGK2. Lipid staining by Oil Red O (red) of NW-MSCs after 21 days of adipogenesis with 10 and 20 μM of AGK2, confirming higher adipogenesis by inhibiting SIRT2.


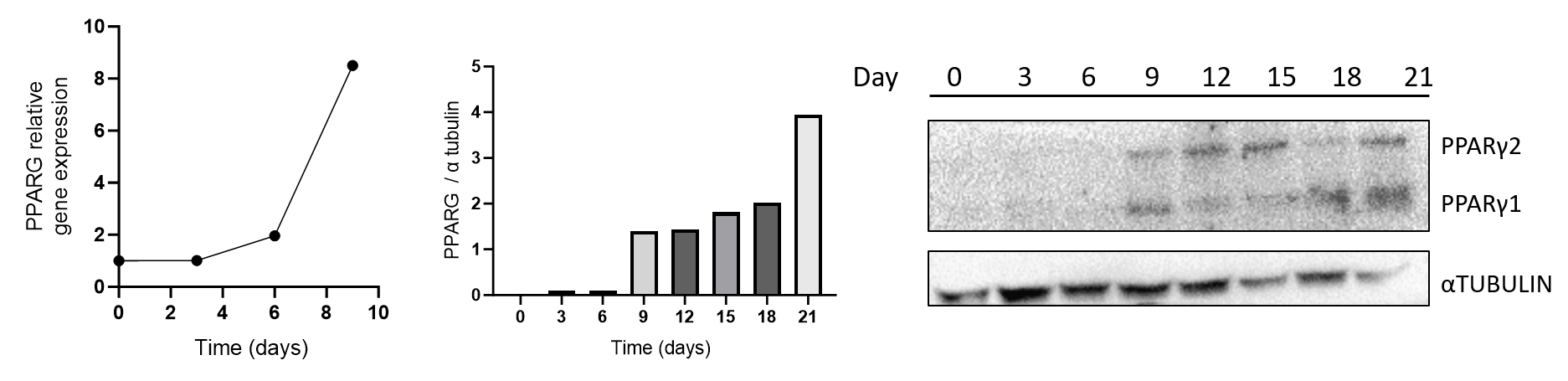


A B

**Supplementary Figure S2. Gene and protein expression of PPARγ during adipogenesis of MSCs.** **A.** PPARG gene expression during 9 days of adipogenesis. **B**. PPARγ1 and PPARγ2 protein expression during 21 days of adipogenesis (n=1 NW-MSCs).


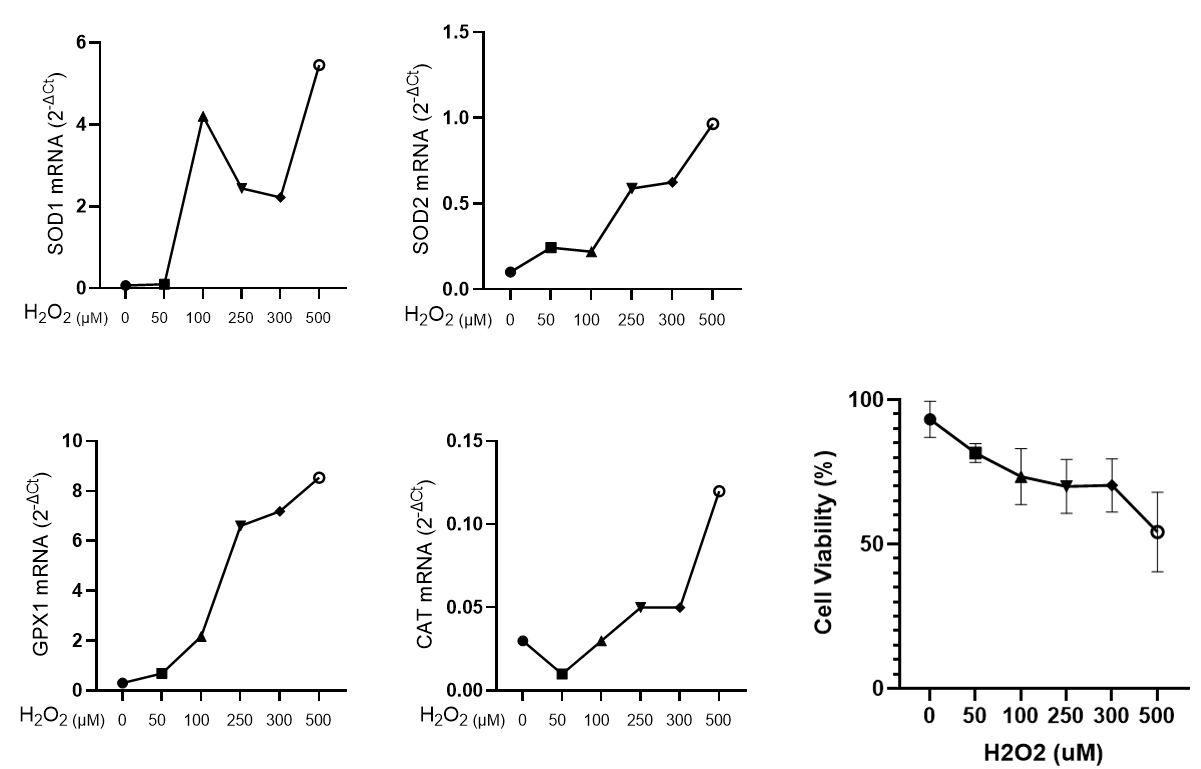


**Supplementary Figure S3. H₂O₂ challenge and viability assays in MSCs.** Concentration-response to H₂O₂ experiments in NW-MSCs for standardization of an oxidative challenge. Left: gene expression of SOD1, SOD2, GPX1 and CAT was assessed after six hours of incubation with H_2_O_2_. Right: cell viability was evaluated with trypan blue in response to H₂O₂.

**
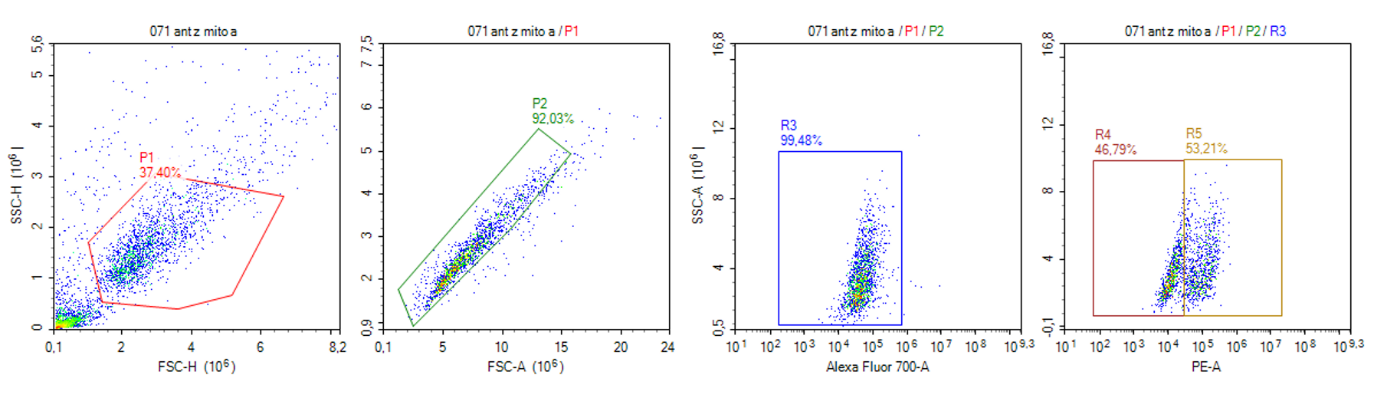
**

**Supplementary Figure S4: Gating strategy for response to H₂O₂, tBHP and antimycin A challenges.** Five thousand events were counted and gated for SSC vs FSC, later exclusion of doublets and viability by ZombieNIR (Alexa Fluor 700).

**
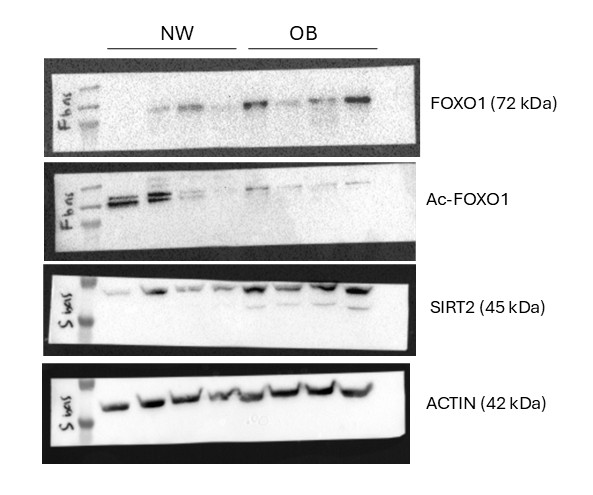
**

**
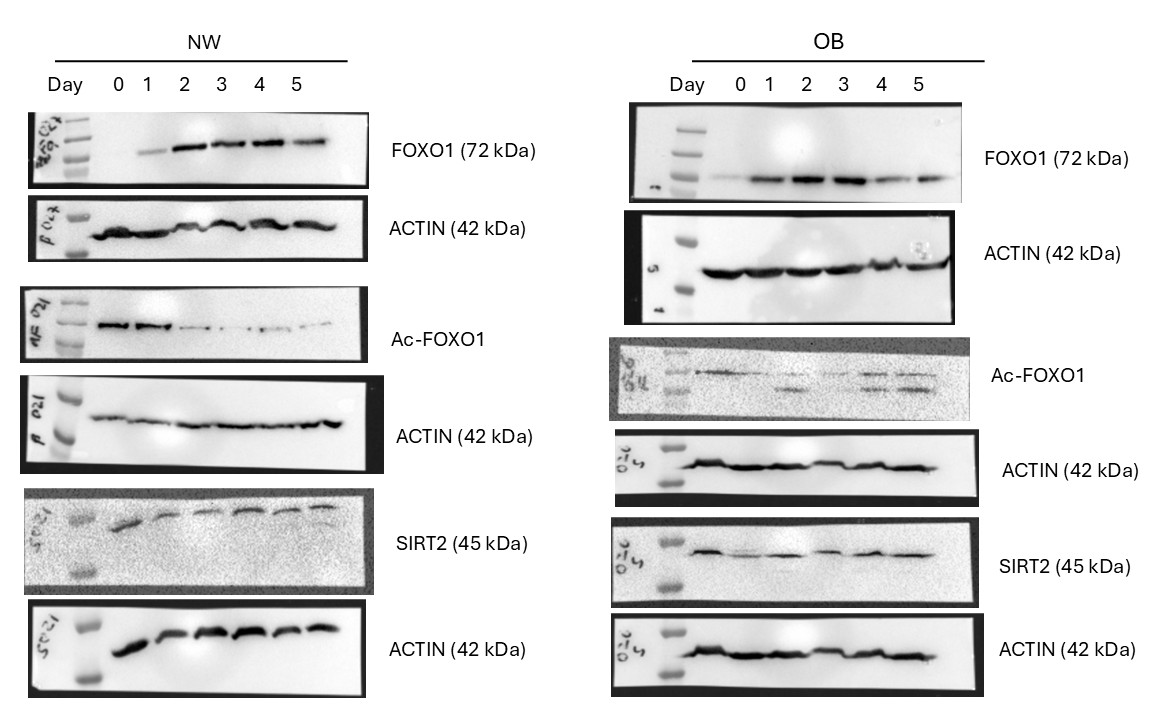
**

*
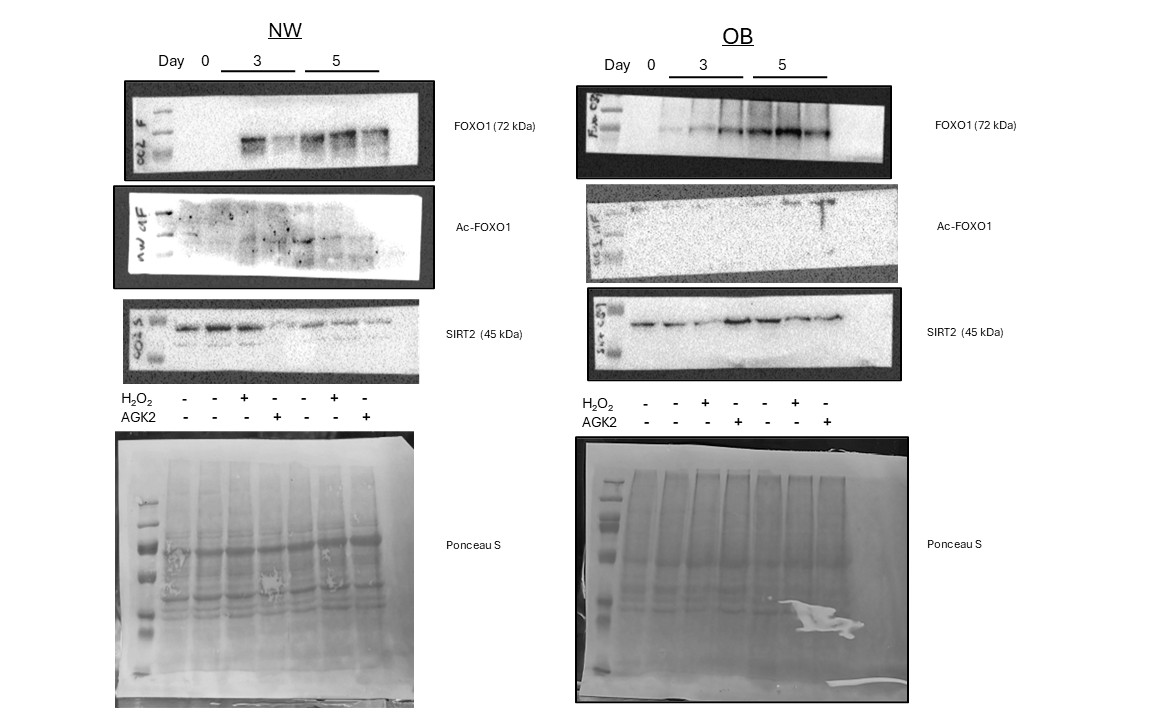
(Continued)*

**Supplementary Figure S5. Original western blot images.** Unprocessed images of complete membranes corresponding to the western blots shown in Figure 1, 2 and 3. Molecular weights are included for FOXO1, SIRT2 and ACTIN. For experiments on Figure 3, membranes were stained with Ponceau S for total protein as reference of protein quantity.

**
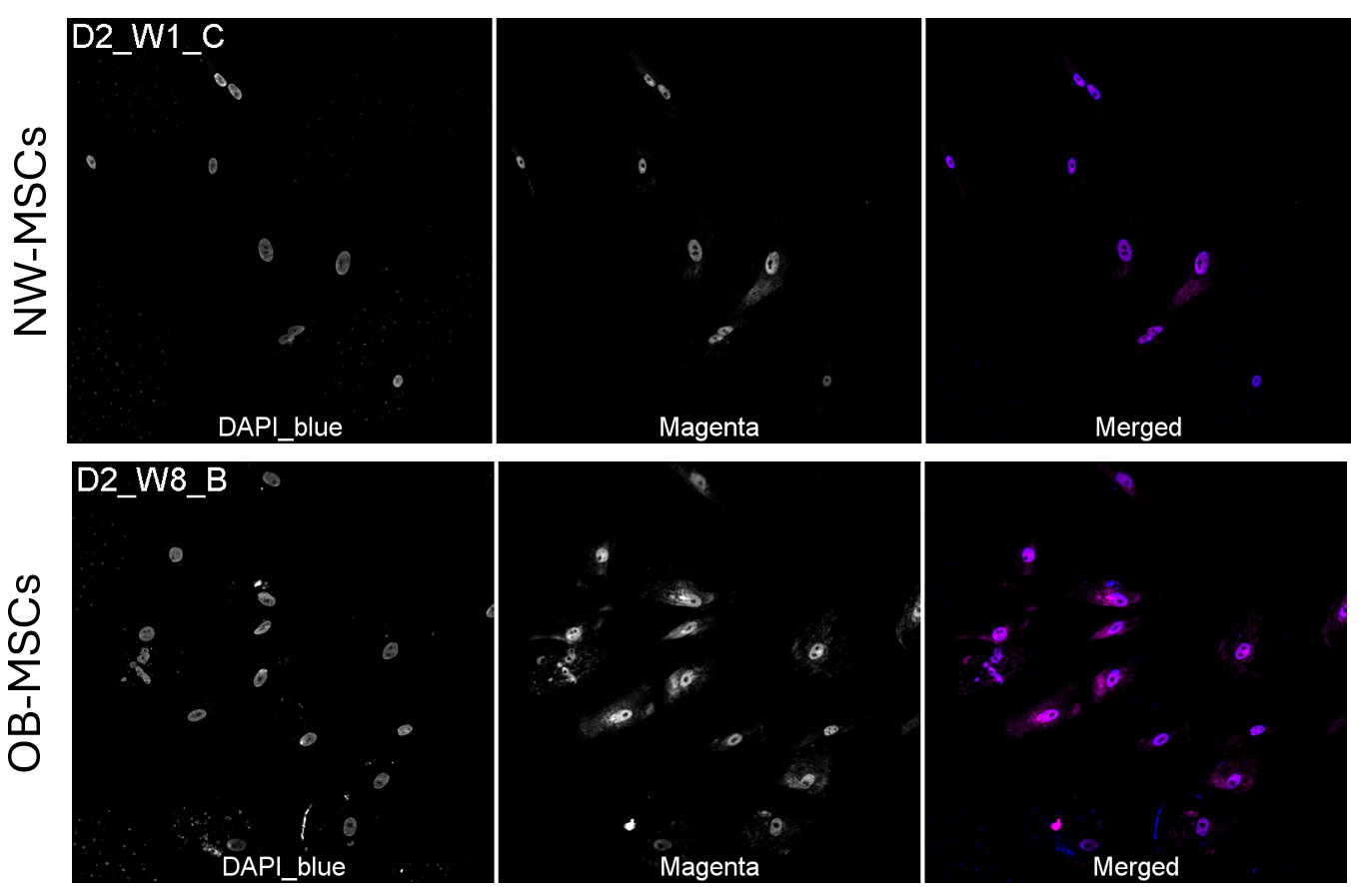
**

**Supplementary Figure S6.** Localization of acetyl-FOXO1 in NW-MSCs and OB-MSCs on day 2. Additional image including a total of 9-15 cells per field (n=5 NW-MSCs and n=5 OB-MSCs).
